# All-Dry Molecular Scale Processing for Shaping Open Pockets with CO_2_ Affinity in a Covalent Organic Framework

**DOI:** 10.1021/cbe.6c00035

**Published:** 2026-04-29

**Authors:** Zhiwen Chen, Yicheng Luo, Jipeng Xu, Ming Zhang, Chunxiao Li, Rundao Chen, Yingwu Luo, Zongbi Bao, Cheng Lian, Junjie Zhao

**Affiliations:** † State Key Laboratory of Chemical Engineering and Low-Carbon Technology, College of Chemical and Biological Engineering, 12377 Zhejiang University, 866 Yuhangtang Rd, Hangzhou 310058, China; ‡ Institute of Zhejiang University-Quzhou, 99 Zheda Rd, Quzhou, Zhejiang 324000, China; § School of Chemistry and Molecular Engineering, 47860 East China University of Science and Technology, Shanghai 200237, China

**Keywords:** covalent organic frameworks, molecular layer deposition, postsynthetic modification, pore engineering, CO_2_ adsorption

## Abstract

Carbon dioxide capture is key to achieving carbon neutrality and addressing climate change. Covalent organic frameworks (COFs) with tailored pore structures have emerged as potential candidates for CO_2_ adsorption. However, previous postsynthetic modification methods often face an inevitable trade-off between increasing adsorption sites and maintaining pore accessibility. Herein, we report an all-dry molecular scale processing (MSP) approach that forms open pockets in COF mesopores by covalently grafting chains with Zn open metal sites and primary amine groups as dual CO_2_-philic sites. The resulting COF not only maintains a BET surface area up to 1180 m^2^ g^–1^ but also enables an ideal selectivity for CO_2_/N_2_ of 153 at 100 kPa, which is almost 10-fold higher than the pristine COF. Mechanistic investigations reveal that micropore filling and strong binding sites contribute to enhanced CO_2_ adsorption. The pore engineering strategy reported here provides essential insights for developing the next generation of adsorbent materials.

## Graphical abstract

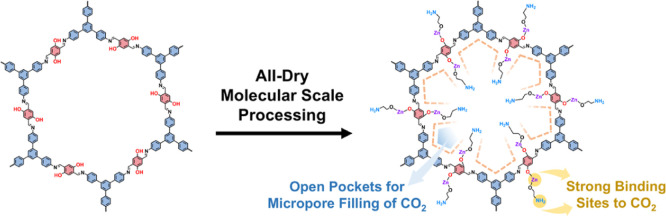

## Introduction

Anthropogenic CO_2_ emissions from fossil fuel combustion represent the dominant driver of global climate change, necessitating advanced materials and separation processes to achieve carbon neutrality.^1–4^ Covalent organic frameworks (COFs) have emerged as potential candidates for CO_2_ capture owing to their large porosity, resistance to moisture, and structural tunability.^5–9^ Decorating COF pores with CO_2_-philic adsorption sites via postsynthetic modification (PSM) could further boost their performance for CO_2_ separation. However, these modification methods often face an inevitable trade-off between the increase of adsorption sites and the remaining porosity and surface area.^10,11^

Various PSM strategies, including click chemistry,^12^ nucleophilic substitution,^13^ ring–opening reactions,^14^ and ion insertion,^15,16^ have successfully incorporated various functional groups into COF pores to promote CO_2_ adsorption. However, previous solution-based methods often have to sacrifice the porosity and specific surface area dramatically in order to introduce a large number of adsorption sites into the COF pores.^17^ Consequently, the limited mass transport poses difficulty in fully removing the unreacted agents, byproducts, and solvents from the modified pores and necessitates extensive pore activation procedures.^18–20^ Additionally, the diffusion of CO_2_ into these modified pores may also be severely hindered. These limitations underscore the urgent need for strategies to achieve the highly preferential adsorption of CO_2_ while preserving a large porosity for COFs.

Herein, we report a unique strategy to create open pockets in COF mesopores using an all-dry molecular scale processing (MSP) method. By covalently grafting short and stiff molecular chains containing both Zn open metal sites and primary amine groups, we generated pentagonal open pockets in the COF pores (Figure 1). The significant potential overlap in such small pockets could lead to micropore filling and determine the low-pressure CO_2_ adsorption.^21^ While MOFs naturally possessing open pockets that exhibit micropore filling behaviors have been reported,^22–24^ so far forming open pockets in as-synthesized COF pores has not yet been explored. In addition to the topological features of these pockets, the Zn open metal sites as Lewis acidic centers^25–28^ and the primary amine end groups^29^ in the grafted ∥–Zn–O–C_2_H_4_NH_2_ chains could also contribute to the enhanced interactions with CO_2_ adsorbates.

**Figure 1:**
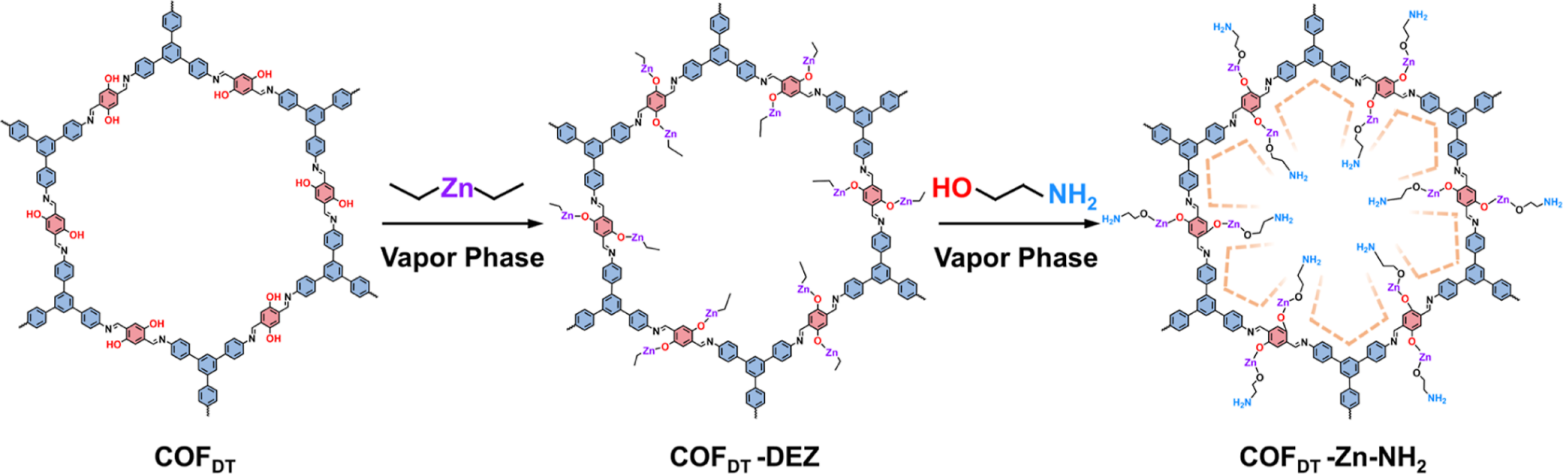
Schematic representation of all-dry MSP for pore engineering in COFs. Sequential self-limiting reactions with diethylzinc (DEZ) and ethanolamine (EA) precursors enable covalent grafting of −Zn–O–C_2_H_4_NH_2_ chains in COF pores, forming open pockets (orange dashed lines) with Zn open metal sites and primary amine groups for selective CO_2_ adsorption.

## Results and Discussion

We chose a mesoporous imine-linked COF (COF_DT_) as our model structure for pore engineering. We synthesized this COF via Schiff-base condensation between 1,3,5-tris­(4-aminophenyl)­benzene (TAPB) and 2,5-dihydroxyterephthalaldehyde (DHTA) (Figure S1). Powder X-ray diffraction (PXRD) reveals the formation of a highly crystalline COF_DT_ with an AA-stacking arrangement (Figure 2a and S3).^30,31^ The characteristic peak at 2.8° corresponds to the (100) plane with a *d*-spacing of 3.2 nm, which agrees with the well-defined lattice fringes and aligned one-dimensional channels found in the transmission electron microscopic (TEM) images (Figure S4).^32–34^ Additional X-ray diffraction peaks at 4.8°, 5.5°, 7.3°, and 9.6° were indexed to the (110), (200), (210), and (220) planes, respectively.

**Figure 2:**
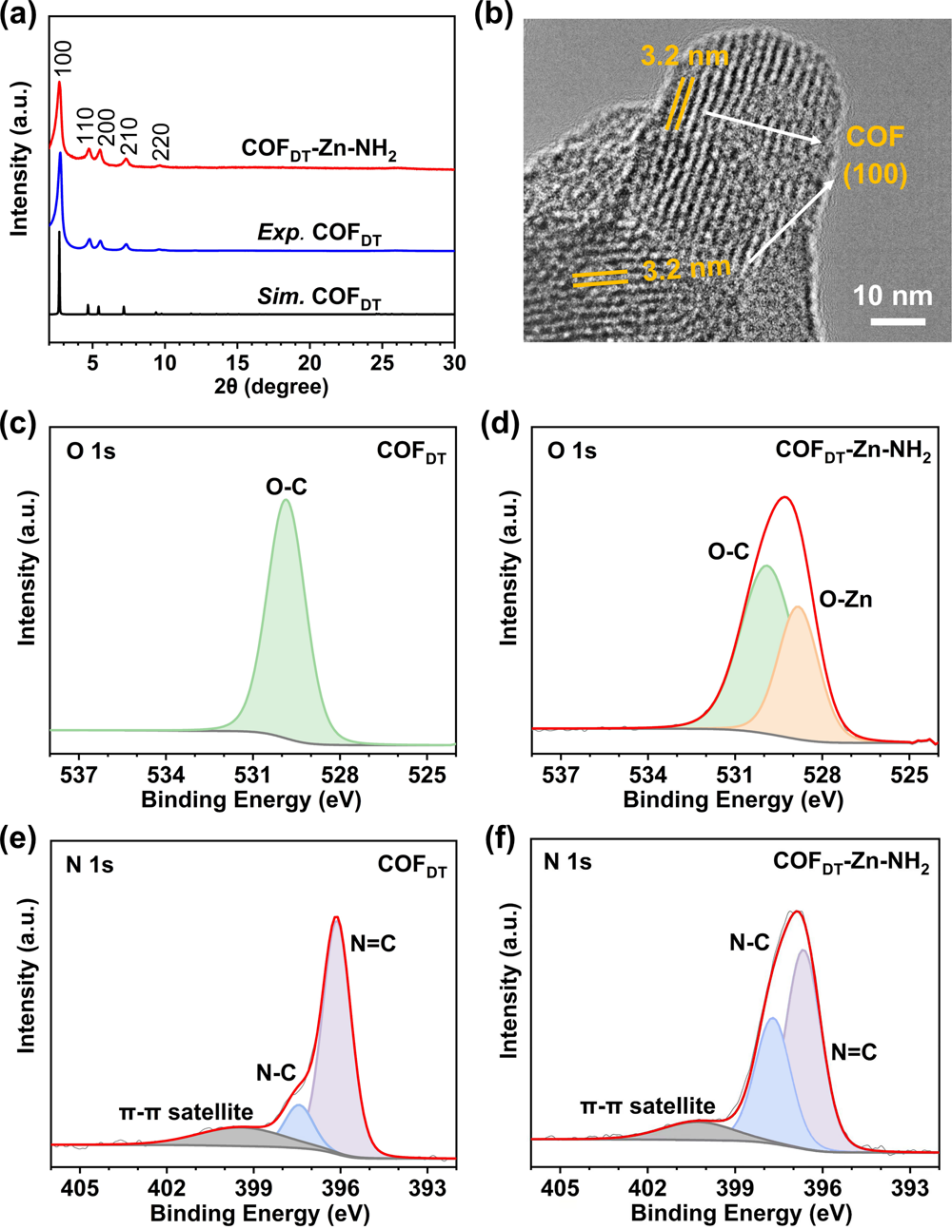
(a) PXRD patterns. (b) HRTEM image of COF_DT_-Zn-NH_2_. O 1s and N 1s XPS spectra for (c, e) COF_DT_ and (d, f) COF_DT_-Zn-NH_2_.

After MSP functionalization, the resulting COF_DT_-Zn-NH_2_ retained high crystallinity (Figure 2a). Scanning electron microscopy (SEM) and TEM images show negligible morphological alteration after MSP functionalization (Figure 2b and S5), while EDS elemental mapping confirms the uniform distribution of Zn and N elements throughout the COF_DT_-Zn-NH_2_ (Figure S6).

We further used X-ray photoelectron spectroscopy (XPS) to analyze the structural changes due to MSP functionalization (Figure S7 and Table S1). The O 1s spectrum of pristine COF_DT_ exhibits a single peak at 529.8 eV, characteristic of the O–C bonds in the phenol groups of COF_DT_ (Figure 2c).^35,36^ In comparison, COF_DT_-Zn-NH_2_ shows an additional O 1s component at 528.8 eV^37,38^ attributed to Zn–O bonds in the ∥–Zn–O–C_2_H_4_NH_2_ moieties (Figure 2d). Inductively coupled plasma mass spectrometry (ICP-MS) reveals a Zn content of 9.75 wt % in COF_DT_-Zn-NH_2_ (Table S2), which corresponds to approximately 5.60 Zn atoms per COF_DT_ pore unit, equivalent to a grafting efficiency of 93.3% with regard to the hydroxyl sites of COF_DT_. Furthermore, N 1s spectra reveal that the peak area ratio between the N–C component (397.3 eV) and the N = C component (396.4 eV) increased dramatically from 0.18 to 0.63 after MSP functionalization (Figure 2e,f and Table S1). Fourier transform infrared (FTIR) spectrum shows N–H stretching at around 3350 cm^–1^, C–H stretching at 2935 and 2866 cm^–1^, C–C vibration at 1386 cm^–1^, and C–O vibration at 1047 cm^–1^ that are associated with the −O–C_2_H_4_NH_2_ moieties in COF_DT_-Zn-NH_2_ (Figure S8).^29–31^

We characterized the COF_DT_ and COF_DT_-Zn-NH_2_ by using N_2_ physisorption at 77 K (Figure 3a). The COF_DT_ exhibited a Brunauer–Emmett–Teller (BET) surface area of 2597 m^2^ g^–1^, substantially higher than the previously reported values.^30,31^ Such a high BET surface area resulted from our optimized synthesis conditions and supercritical CO_2_ activation protocols that ensured complete pore evacuation. After MSP functionalization, COF_DT_-Zn-NH_2_ maintained a BET surface area as high as 1180 m^2^ g^–1^.

**Figure 3:**
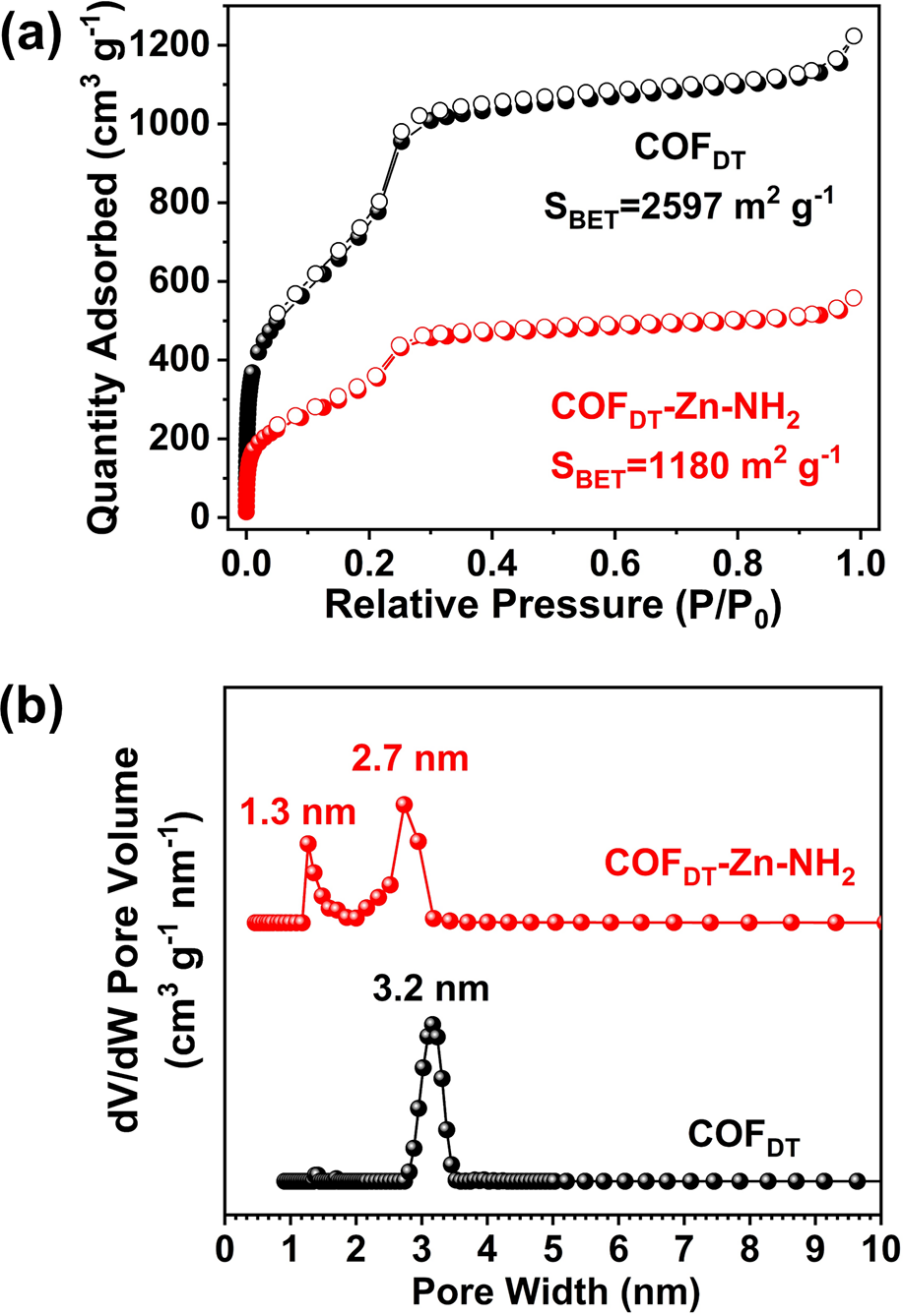
(a) N_2_ adsorption isotherms measured at 77 K and (b) pore size distributions of COF_DT_ and COF_DT_-Zn-NH_2_.

We further analyzed the pore size distribution based on the N_2_ isotherm using nonlocal density functional theory (NLDFT) (Figure 3b). Pristine COF_DT_ exhibited a sharp unimodal distribution centered at 3.2 nm, confirming its highly ordered mesoporous structure. After MSP modification, COF_DT_-Zn-NH_2_ showed a complete transformation of the original 3.2 nm mesopores into a bimodal distribution with distinct pore sizes centered at 2.7 and 1.3 nm. The rise of the small pores with a diameter of 1.3 nm is ascribed to the pentagonal open pockets formed by the ∥–Zn–O–C_2_H_4_NH_2_ chains and the COF side walls.

We further investigated the adsorptive separation of CO_2_/N_2_ by COF_DT_ and COF_DT_-Zn-NH_2_. Adsorption isotherms for COF_DT_ at 298 K (Figure 4a) reveal a gradual increase of the levels of CO_2_ and N_2_ uptake with pressure. In contrast, with the open pockets formed in the COF pores, COF_DT_-Zn-NH_2_ exhibits a steep increase of CO_2_ uptake at the pressure range below 2.54 kPa, which is a typical micropore filling behavior and indicates significantly enhanced CO_2_ affinity.^23,39,40^ We found that the CO_2_ adsorption capacity of COF_DT_-Zn-NH_2_ at 100 kPa (32.5 cm^3^ g^–1^) is 42% higher than the pristine COF_DT_. Additionally, N_2_ uptake of COF_DT_-Zn-NH_2_ at 100 kPa was suppressed to merely 0.14 cm^3^ g^–1^, approximately 12-fold lower than the pristine COF_DT_.

**Figure 4:**
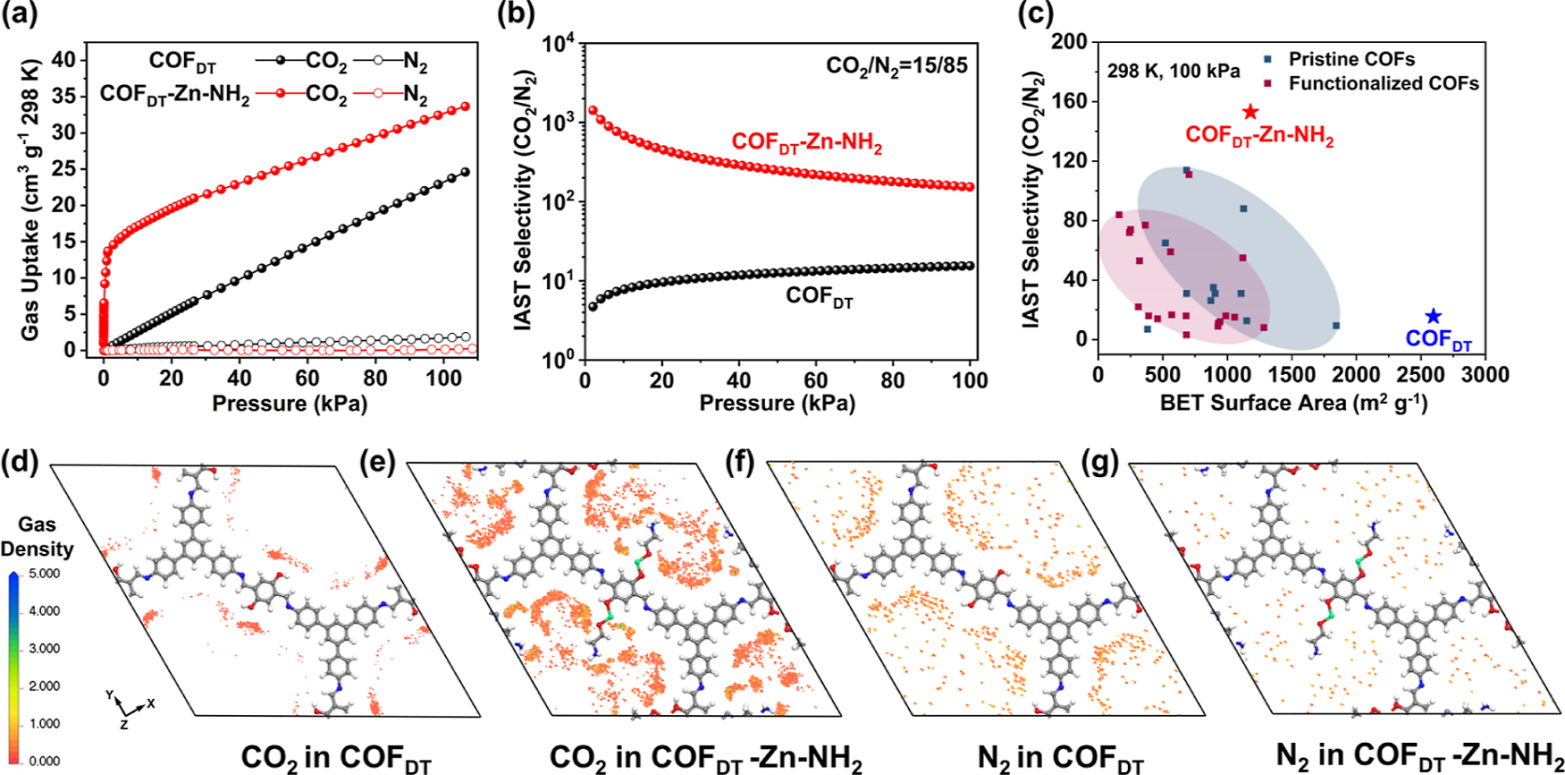
(a) CO_2_ and N_2_ adsorption isotherms. (b) IAST selectivity of CO_2_/N_2_ (15:85, v/v) gas mixture for COF_DT_ and COF_DT_-Zn-NH_2_ at 298 K. (c) Comparison of BET surface area and IAST selectivity for COF_DT_-Zn-NH_2_ in this work with the COFs previously reported in literature. (d-g) Density distributions of N_2_ and CO_2_ molecules at 298 K and 100 kPa in COF_DT_ and COF_DT_-Zn-NH_2_. (Gray, white, blue, red, and green atoms represent C, H, N, O, and Zn, respectively.).

We evaluated CO_2_/N_2_ separation selectivity for simulated flue gas composition (CO_2_/N_2_ = 15:85, v/v) at 298 K using ideal adsorbed solution theory (IAST) (Figure 4b) .^12,13^ COF_DT_-Zn-NH_2_ achieved a remarkable ideal selectivity of CO_2_/N_2_ over 1000 at pressures below 3.02 kPa. The ideal selectivity obtained by COF_DT_-Zn-NH_2_ at 100 kPa also reached 153, reflecting almost 10-fold enhancement over the pristine COF_DT_ (15.5). Compared with the COF materials reported previously, our COF_DT_-Zn-NH_2_ exhibits an unprecedented ideal selectivity of CO_2_/N_2_ while maintaining a BET surface area of over 1000 m^2^ g^–1^ (Figure 4c and Table S3). Such a high surface area facilitates the rapid diffusion of gas molecules within the pores, thereby enhancing the overall mass transfer efficiency.^41^

We calculated the isosteric heat of adsorption (*Q*_st_) using CO_2_ adsorption data at 273 and 298 K (Figure S9).^42,43^ COF_DT_-Zn-NH_2_ shows a higher initial *Q*_st_ (25.1 kJ mol^–1^) than the pristine COF_DT_ (20.0 kJ mol^–1^). This enhancement stems primarily from the Zn open metal sites and primary amine groups that we introduced into the COF pores. Density functional theory (DFT) simulation results (Figure S10) also reveal that the binding energy of CO_2_ substantially changes from −4.90 kJ mol^–1^ to −10.92 kJ mol^–1^ due to the MSP functionalization, further confirming the stronger CO_2_ affinity with COF_DT_-Zn-NH_2_.

To further understand the molecular-level adsorption mechanism, we conducted a grand canonical Monte Carlo (GCMC) simulations. Simulated adsorption isotherms at 298 K closely match the experimental results (Figure S11). CO_2_ density distribution in COF_DT_ at 298 K and 100 kPa shows adsorption primarily near the imine bonds and phenol groups of the pristine COF_DT_ (Figure 4d). In comparison, densely adsorbed CO_2_ molecules were found to accumulate in the pentagonal open pockets formed in COF_DT_-Zn-NH_2_ (Figure 4e), consistent with the micropore filling observed in our experiments. Within the open pockets, preferential CO_2_ binding to the Zn open metal sites can be attributed to Lewis acid–base coordination.^26,44^ Additionally, enhanced CO_2_ density near the primary amine groups arises from hydrogen bonding and dipole–quadrupole interactions.^29,45^ Figure 4f,g shows lower N_2_ density in the COF_DT_-Zn-NH_2_ pores than in the COF_DT_ pores, which agrees with the N_2_ isotherms measured at 298 K and can be explained by the weakened N_2_ binding energy (Figure S10) found in our DFT simulation.

Finally, we performed breakthrough tests to further evaluate the separation of mixed CO_2_/N_2_ (15:85, v/v) at 298 K. Figure S12 shows that N_2_, as a weakly adsorbed component, rapidly eluted from the column, whereas CO_2_ required a prolonged period (29.3 min) to breakthrough. The dynamic adsorption capacity of CO_2_ calculated from the breakthrough curve is 15.4 cm^3^ g^–1^, which is comparable to the uptake of pure CO_2_ at 15 kPa (16.9 cm^3^ g^–1^) found in the adsorption isotherm.

## Conclusion

In conclusion, we have developed an all-dry MSP approach to build open pockets within the COF pores. We used vapor-phase diethylzinc and ethanolamine precursors to covalently graft ∥–Zn–O–C_2_H_4_NH_2_ chains to the COF pore walls. These chains together with the COF pore walls form pentagonal open pockets in the original 3.2 nm mesopores, leading to a bimodal distribution with distinct pore sizes of 2.7 and 1.3 nm. Both adsorption measurements and computational simulations confirm the micropore filling of CO_2_ in the open pockets. We also found that the Zn open metal sites and amine groups of the grafted chains exhibit preferential CO_2_ binding, while the modified pore structure suppresses N_2_ adsorption. Consequently, the resulting COF structure achieves an exceptional ideal selectivity of CO_2_/N_2_ (15:85, v/v) of 153 at 100 kPa, which is an almost 10-fold increase compared to the pristine COF and surpasses any prior reports. Unlike previous solution-based pore modification methods, our all-dry MSP circumvents the inevitable diffusional constraints and allows the maintenance of the COF BET surface area as high as 1180 m^2^ g^–1^. Our pore engineering strategy offers insights for future development of high-performance materials for carbon capture and gas separation.

## Supplementary Material



## Notes

Experimental details, characterization, gas adsorption test, supplementary characterization results, synthesis route for COF_DT_; pore functionalization in COF_DT_ via MSP; top and side views of the structure of COF_DT_; TEM and HRTEM images of COF_DT_; SEM images of pristine COF_DT_ and COF_DT_-Zn-NH_2_ after MSP functionalization; TEM image and EDS mapping images of COF_DT_-Zn-NH_2_; XPS survey spectra of COF_DT_ and COF_DT_-Zn-NH_2_; FTIR spectra of TAPB, DHTA, COF_DT_, and COF_DT_-Zn-NH_2_; XPS binding energies and peak area ratios of COF_DT_ and COF_DT_-Zn-NH_2_; elemental composition of COF_DT_ and COF_DT_-Zn-NH_2_ determined by XPS and ICP-MS analyses; isosteric heat of CO_2_ adsorption for COF_DT_ and COF_DT_-Zn-NH_2_; comparison of IAST selectivity between MSP-functionalized COF_DT_-Zn-NH_2_ in this work and COF materials reported previously in literature; DFT-optimized stable adsorption configurations and binding energies of CO_2_ and N_2_ molecules on representative structural units of COF_DT_ and COF_DT_-Zn-NH_2_; CO_2_ and N_2_ adsorption isotherms of COF_DT_ and COF_DT_-Zn-NH_2_ at 298 K obtained from GCMC simulations; and breakthrough curves of a CO_2_/N_2_ mixture at 298 K for COF_DT_ and COF_DT_-Zn-NH_2_ (PDF)
