# Supplementary material for: All-Dry Molecular Scale Processing for Shaping Open Pockets with CO2 Affinity in a Covalent Organic Framework
Source: Chem Bio Eng. 2026 Apr 29;3(7):650–5. doi: 10.1021/cbe.6c00035 (PMC13403142; doi:10.1021/cbe.6c00035)
Supplement: Supplementary file 1 [file be6c00035_si_001.pdf]

Supporting Information

All-Dry Molecular Scale Processing for Shaping Open Pockets with CO_2 Affinity in a Covalent Organic Framework

Zhiwen Chen[a,b,#], Yicheng Luo [a,b,#], Jipeng Xu[c], Ming Zhang[a,b], Chunxiao Li[a,b], Rundao Chen[a], Yingwu Luo[a], Zongbi Bao[a,b], Cheng Lian[c,*] and Junjie Zhao[a,b,*]

a State Key Laboratory of Chemical Engineering and Low-Carbon Technology, College of Chemical and Biological Engineering, Zhejiang University, 866 Yuhangtang Rd, Hangzhou 310058, China
Email: junjiezhao@zju.edu.cn

b Institute of Zhejiang University - Quzhou, 99 Zheda Rd, Quzhou, Zhejiang 324000, China

c School of Chemistry and Molecular Engineering, East China University of Science and Technology, Shanghai 200237, China
Email: liancheng@ecust.edu.cn

* Corresponding author

# These authors contributed equally to this work

Experimental Methods

Chemicals

2,5-Dihydroxyterephthalaldehyde (DHTA, 98%), 1,3,5-tris(4-aminophenyl)benzene (TAPB, 98%), and ethanolamine (EA, ≥99.7%) were purchased from Shanghai Aladdin Biochemical Technology Co., Ltd. Acetic acid (AcOH, ≥99.5%), 1,2-dichlorobenzene (*o*-DCB, ≥99.0%), 1-butanol (*n*-BuOH, ≥99.5%), tetrahydrofuran (THF, ≥99.0%), N,N-dimethylformamide (DMF, ≥99.5%), acetone (≥99.5%), and ethanol (≥99.7%) were purchased from Sinopharm Chemical Reagent Co., Ltd. Diethylzinc (DEZ, 99.999%) was obtained from Nanjing Ai Mou Yuan Scientific Equipment Co., Ltd. All chemicals were used as received without further purification.

Synthesis of COF_{DT}

A Schlenk tube was charged with DHTA (249 mg, 1.5 mmol) and TAPB (351 mg, 1.0 mmol). A solvent mixture of *o*-DCB (9 mL) and *n*-BuOH (9 mL) was added, and the mixture was sonicated for 10 min to ensure thorough dispersion of the reactants. AcOH (3 mL, 6 M aqueous solution) was added as a catalyst. The reaction mixture underwent three freeze-pump-thaw cycles to remove oxygen, was sealed under vacuum, and heated at 120 °C for 3 days (**Figure S1**). The resulting orange solid was collected by filtration and purified through sequential hot washing with DMF (200 mL, 70 °C), THF (200 mL, 60 °C), and acetone (200 mL, 50 °C), with each washing step conducted for 30 min followed by hot filtration. The purified powder was activated by supercritical CO_2 extraction (80 °C, 12 MPa) to afford orange COF_{DT} powder (453 mg, 83% yield).

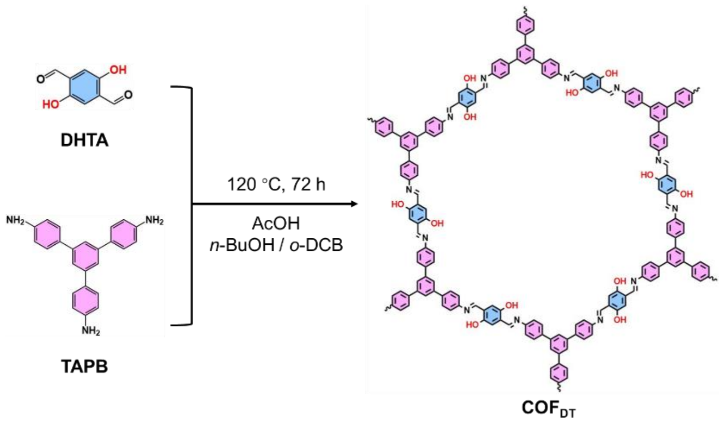

Figure S1. Synthesis route for COF_{DT}.

Pore Functionalization *via* Molecular Scale Processing (MSP)

The all-dry MSP method was carried out in a viscous-flow atomic layer deposition reactor. Diethylzinc (DEZ) and ethanolamine (EA) were used as the metal precursor and amine source, respectively. High-purity N_2 (99.999%) was used as the inert carrier and purge gas. Prior to the MSP process, COF_{DT} powder (100 mg) was placed in a custom-made stainless-steel holder and degassed in the reaction chamber at 120 °C for 12 h to remove adsorbed water molecules.

In a typical MSP supercycle, DEZ was first dosed for 0.5 s and held in the chamber for 60 s to allow diffusion and reaction in the COF_{DT} pores. The chamber was then purged with N_2 for 120 s at 20 sccm to remove the byproducts and unreacted precursors. These dose/hold/purge steps were repeated 10 times for DEZ, followed by an extended N_2 purge (1800 s) under vacuum to thoroughly evacuate the chamber before introducing EA. The EA source was heated to 60 °C, and the dose (1 s)/hold (90 s)/purge (120 s) sequence for EA was repeated 10 times to complete one full MSP supercycle (**Figure S2**). The resulting functionalized COF material was designated as COF_{DT}-Zn-NH_2.

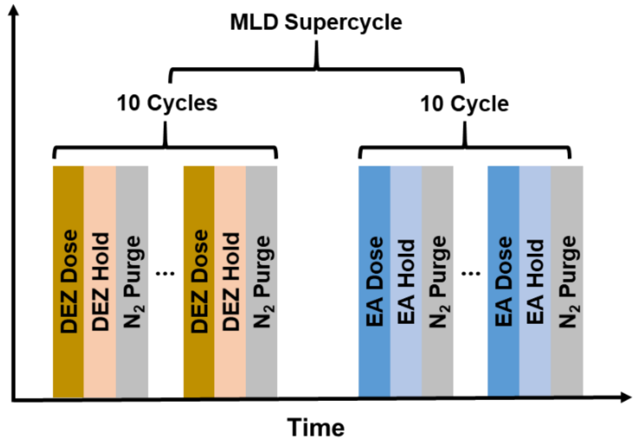

Figure S2. Schematic illustration of pore functionalization in COF_{DT} via MSP.

Characterizations

Powder X-ray diffraction (PXRD) patterns were recorded on a Bruker D8 ADVANCE with Cu Kα radiation (λ=1.5406 Å). Morphological and structural characterizations were conducted using field emission scanning electron microscopy (FESEM, Hitachi SU8010) at an accelerating voltage of 3 kV after 120 s Pt coating, and transmission electron microscopy (TEM, HITACHI HT7820) at 120 kV with high-resolution imaging (HRTEM,

JEOL JEM-F200) at 200 kV. Samples for TEM were prepared by dispersing powders in ethanol via sonication (30 min) before drop-casting onto carbon-coated copper grids. Energy-dispersive X-ray spectroscopy (EDS, Oxford X-max 80) was performed at 15 kV for elemental mapping and semi-quantitative analysis. Fourier-transform infrared (FTIR) spectra were recorded on a ThermoFischer iS50 spectrometer using KBr pellets in transmission mode (400-4000 cm^{-1}). X-ray photoelectron spectroscopy (XPS, Thermo Scientific ESALAB250Xi) with monochromatic Al-Kα source was employed for surface chemical state analysis, with all spectra calibrated to C 1s (284.8 eV). Nitrogen adsorption-desorption isotherms were measured on a Micromeritics ASAP 2460 analyzer at 77 K after degassing samples at 120 °C under vacuum for 12 h. Brunauer-Emmett-Teller (BET) method was applied to calculate specific surface areas, while pore size distributions were derived using non-local density functional theory (NLDFT) models. Zinc content was quantified by inductively coupled plasma mass spectrometry (ICP-MS, Agilent 7800) following complete digestion in concentrated nitric acid.

Gas Adsorption

Gas adsorption isotherms were collected using a Micromeritics ASAP 2460 surface area and porosity analyzer. Prior to measurements, approximately 50 mg of sample was degassed at 120 °C under vacuum for 12 h to completely remove adsorbed species from the pore channels. CO_2 and N_2 adsorption isotherms were measured at both 273 K and 298 K in the pressure range of 0-100 kPa.

To evaluate the CO_2/N_2 separation performance, the experimental adsorption data were fitted using a dual-site Langmuir-Freundlich model (Equation S1):

$$q = q_{m1} \times \frac{b_1 \times p^{1/n_1}}{1+b_1 \times p^{1/n_1}} + q_{m2} \times \frac{b_2 \times p^{1/n_2}}{1+b_2 \times p^{1/n_2}} \quad (1)$$

where q is the adsorption amount (mmol g^{-1}), p is the equilibrium pressure (kPa), q_{m1} and q_{m2} are the saturation capacities for sites 1 and 2 (mmol g^{-1}), b_1 and b_2 are the affinity coefficients (kPa^{-1}), and n_1 and n_2 represent deviations from surface homogeneity.

The selectivity, S, for CO_2/N_2 (15:85 v/v) was calculated based on the Ideal Adsorbed Solution Theory (IAST)[1,2] using Equation S2:

$$S = \frac{x_1/x_2}{y_1/y_2} \quad (2)$$

where x_1 and x_2 are the mole fractions in the adsorbed phase, and y_1 and y_2 are the mole fractions in the gas phase for CO_2 and N_2, respectively.

The isosteric heat of adsorption, Q_{st}, was calculated using the virial equation[3,4] (Equation S3) based on CO_2 adsorption isotherms at 273 K and 298 K:

$$\ln p = \ln N + \frac{1}{T}\sum_{i=0}^{m} a_i N^i + \sum_{i=0}^{n} b_i N^i \tag{3}$$

where p is the pressure (kPa), N is the adsorption amount (mmol g^{-1}), T is the absolute temperature (K), and a_i and b_i are virial coefficients with $m \leq 6$ and $n \leq 3$.

The Q_{st} values were then determined using Equation S4:

$$Q_{st} = -R\sum_{i=0}^{m} a_i\, N^i \tag{4}$$

where R is the universal gas constant (8.314 J mol^{-1} K^{-1}).

Breakthrough Tests

The adsorption breakthrough experiment was conducted at 298 K and 100 kPa. Approximately 300 mg of the sample was vacuum-activated at 120 °C for 12 h and then packed into an adsorption column with an inner diameter of 4.6 mm and a length of 10 cm. The column packing was conducted in a glovebox filled with Ar. After the column was installed, the gas line was purged with helium at a flow rate of 1 mL min^{-1} for 2-3 h. Subsequently, the helium flow was stopped, and the mixed gas was introduced at 1 mL min^{-1} to initiate the breakthrough test. The outlet concentrations of He, CO_2, and N_2 were monitored by mass spectrometry (Hiden HPR-20 R&D). The dead time of the system was determined as the point at which the helium concentration began to decline, and the dead volume was calculated accordingly.

The adsorption capacity of gas in the breakthrough experiments can be calculated by the following Equation S5：

$$q_i = \frac{(Ft - V_{dead})c_{0,i} - \int_0^t Fc_{e,i}\mathrm{dt}}{m} \tag{5}$$

where q_i is the adsorption capacity of gas i (mmol g^{-1}), F is the total flow rate (mL min^{-1}), t is the adsorption time (min), V_{dead} is the dead volume of the system (mL), $c_{0,i}$ is the inlet concentration of gas i (mol L^{-1}), $c_{e,i}$ is the effluent concentration of gas i (mol L^{-1}), and m is the mass of the adsorbents (g).

Density Functional Theory (DFT) Calculations

DFT calculations were performed using the Gaussian software package[5] to examine gas-framework interactions. Adsorption configurations of CO_2 and N_2 on representative structural units of pristine COF_{DT} and COF_{DT}-Zn-NH_2 were geometrically optimized using the Perdew-Burke-Ernzerhof 0 (PBE0) functional[6,7] incorporating D3(BJ) dispersion correction[8]. The LANL2DZ effective core potential[9–11] was applied for Zn atoms, while the 6-311G* basis set[12] was employed for all other elements (H, C, N, O). Binding energies were rigorously calculated with Counterpoise correction to eliminate Basis Set Superposition Error (BSSE)[13], enabling accurate quantification of interaction strengths before and after MSP functionalization.

Grand Canonical Monte Carlo (GCMC) Simulations

GCMC simulations[14] were conducted using the Sorption module in Materials Studio employing the COMPASS force field[15] to examine CO_2 and N_2 adsorption in pristine COF_{DT} and COF_{DT}-Zn-NH_2. Simulations were performed at 298 K over pressure ranges from 1.013 to 101.3 kPa, utilizing 1×10^5 equilibration and 1×10^5 production steps to ensure configurational sampling adequacy. Non-bonded interactions were truncated at a cutoff distance of 9.5 Å, with long-range electrostatic interactions treated using the Ewald summation method[16,17] and *van der Waals* interactions handled via the Group-based approach[18].

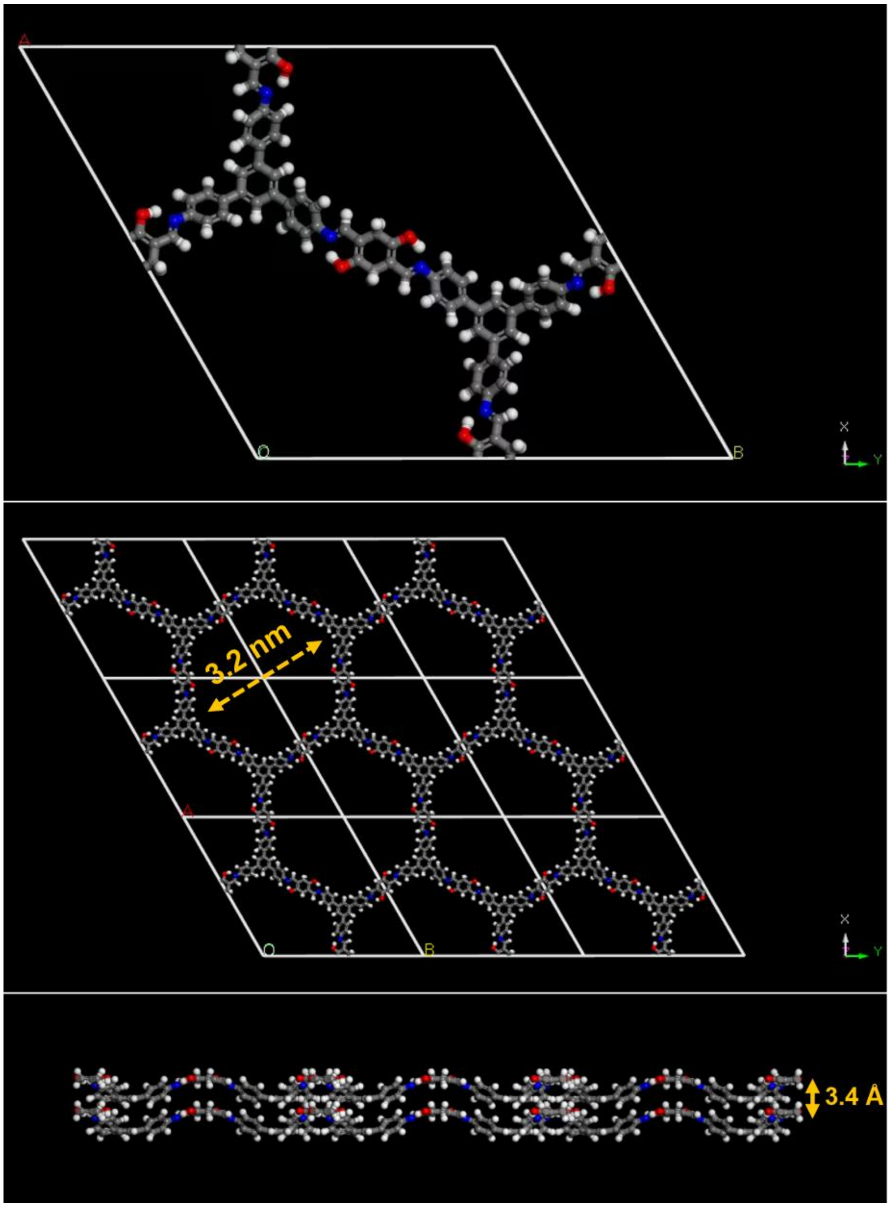

Figure S3. Top-view and side-view structure of COF_{DT}.

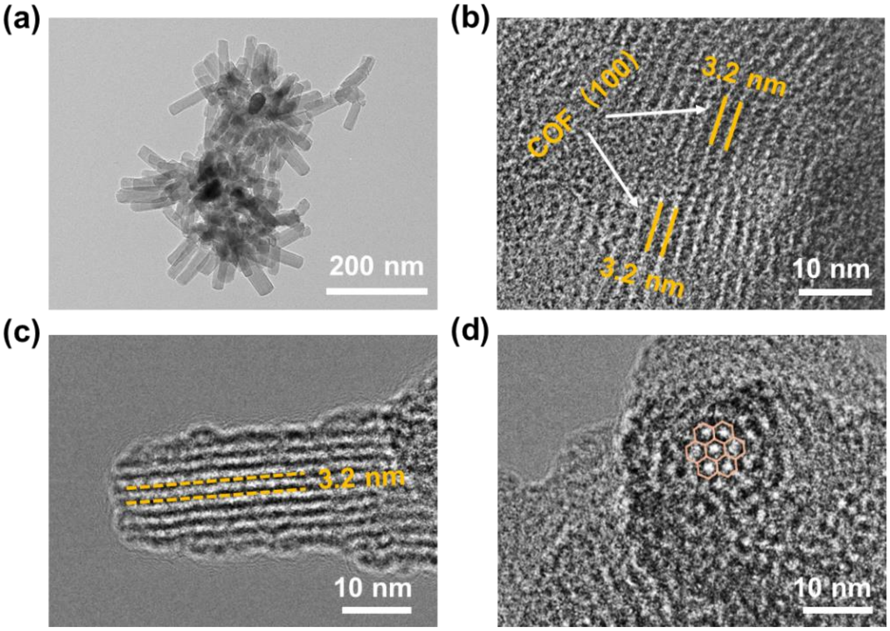

Figure S4. (a) TEM and (b-d) HRTEM images of COF_{DT}.

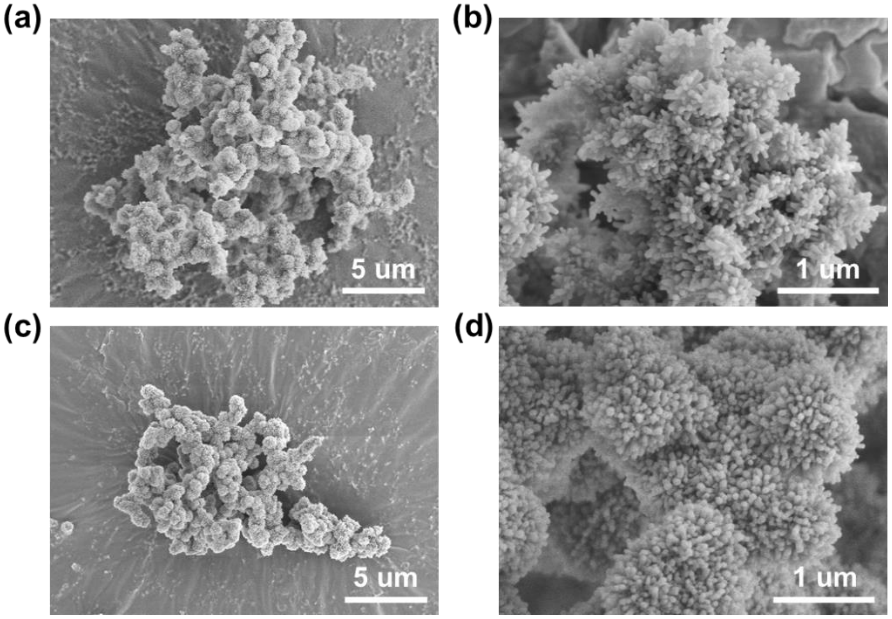

Figure S5. SEM images of (a, b) pristine COF_{DT} and (c, d) COF_{DT}-Zn-NH_2 after MSP functionalization.

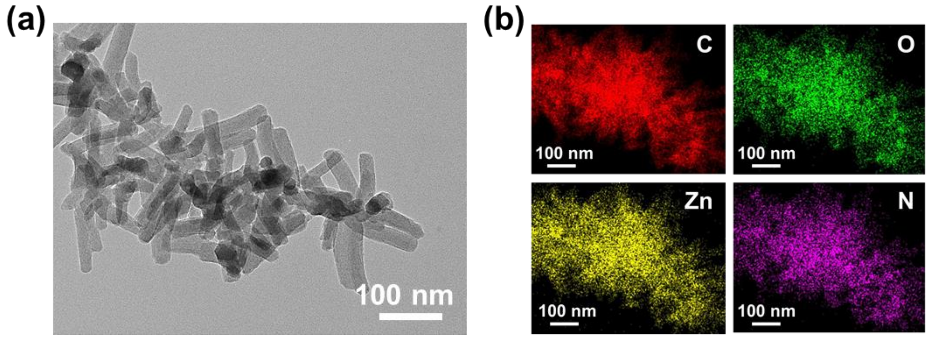

Figure S6. (a) TEM image and (b) EDS mapping images of COF_{DT}-Zn-NH_2. EDS analysis revealed that the C, O, Zn, and N contents of COF_{DT}-Zn-NH_2 are 73.6 wt.%, 7.6 wt.%, 12.8 wt.%, and 6.0 wt.%, respectively.

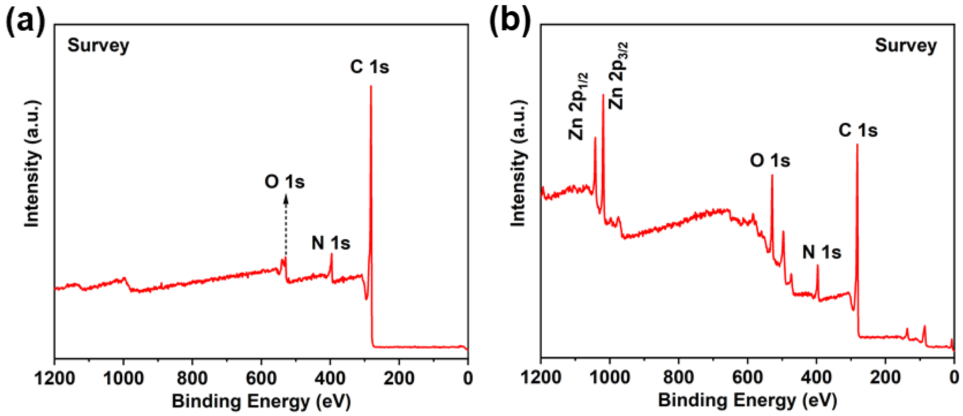

Figure S7. XPS survey spectra of (a) COF_{DT} and (b) COF_{DT}-Zn-NH_2.

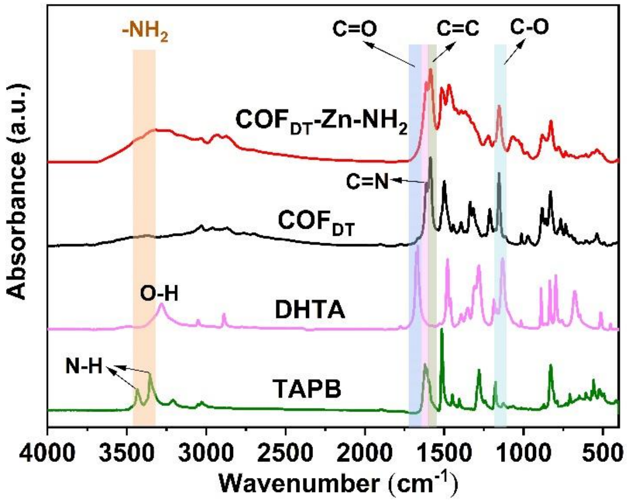

Figure S8. FTIR spectra of TAPB, DHTA, COF_{DT} and COF_{DT}-Zn-NH_2.

Table S1. XPS binding energies and peak area ratios of COF_{DT} and COF_{DT}-Zn-NH_2.

Samples	Binding Energy (eV)				A_{O-Zn}/A_{O-C} [a]	$A_{N-C}/A_{N=C}$ [a]
	O-C	O-Zn	N=C	N-C		
COF_{DT}	529.8	-	396.1	397.2	-	0.18
COF_{DT}-Zn-NH_2	529.9	528.8	396.4	397.3	0.60	0.63

[a] Determined from XPS peak deconvolution and integration.

Table S2. Elemental composition of COF_{DT} and COF_{DT}-Zn-NH_2 determined by XPS and ICP-MS analyses.

Samples	Elemental Composition (wt. %)				
	C	N	O	Zn	Zn [b]
COF_{DT}	86.93	5.78	7.29	-	-
COF_{DT}-Zn-NH_2	73.11	7.76	12.02	7.11	9.75

[b] The Zn values in this column from ICP-MS; all other data from XPS.

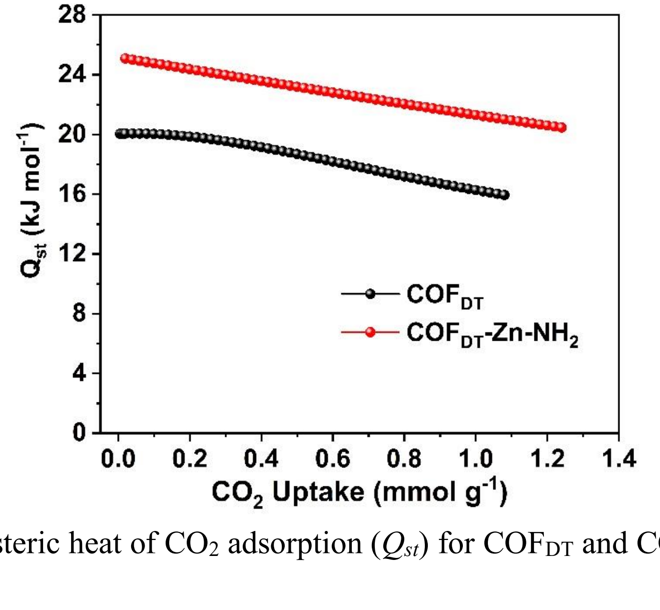

Figure S9. Isosteric heat of CO_2 adsorption (Q_{st}) for COF_{DT} and COF_{DT}-Zn-NH_2.

Table S3. Comparison of IAST selectivity between MSP-functionalized COF_{DT}-Zn-NH_2 in this work and the COF materials reported previously in literature.

Materials	BET surface area (cm^2 g^{-1})	CO_2 Uptake [a] (cm^3 g^{-1})	Q_{st} (kJ mol^{-1})	CO_2/N_2 IAST Selectivity [b]	Reference
NUS-2	697	51.7	38.0	41.2	19
ACOF-1	872	29.1	20.5	26.3	
COF-300	1150	23.5	18.2	12.6	
TAPB-PDA	1845	14.5	24.5	9.40	
COF-LZU1	904	28.0	22.4	31.0	
TpPa-1	684	44.8	44.0	114	
AB-COF	1125	37.6	29.7	88.0	20
Li@AB-COF	988	32.0	32.2	16.0	
Zn@AB-COF	1120	40.1	31.8	55.0	
ATFG-COF	520	53.8	33.3	65.0	
Li@ATFG-COF	242	41.7	34.9	72.0	
Zn@ATFG-COF	162	29.8	42.6	84.0	
$[HC{\equiv}C]_{50}$-H_2P-COF	683	14.6	16.5	3.20	21
$[EtNH_2]_{50}$-H_2P-COF	568	37.7	20.9	16.7	
$[HO]_{100\%}$-H_2P-COF	1284	19.7	36.4	8.00	22
$[HO_2C]_{100\%}$-H_2P-COF	364	42.8	43.5	77.0	
BBO-COF-1	891.0	50.7	30.2	35.0	23
BBO-COF-2	1106	33.8	27.8	31.0	
$[HO]_{25\%}$-TAPH-COFs	927	18.0	30.9	9	24
$[HO]_{50\%}$-TAPH-COFs	930	20.8	28.2	11	
$[HO]_{75\%}$-TAPH-COFs	944	21.4	28.3	12	
$[HO]_{100\%}$-TAPH-COFs	1056	21.4	31.1	15	
$[N{=}N]_{25\%}$-TAPH-COFs	702	64.8	43.4	111	
$[N{=}N]_{50\%}$-TAPH-COFs	560	37.7	36.0	59	
$[N{=}N]_{75\%}$-TAPH-COFs	320	24.8	31.0	53	
$[N{=}N]_{100\%}$-TAPH-COFs	250	22.0	30.7	74	
$[C{=}C]_{25\%}$-TAPH-COFs	680	22.5	30.3	16	
$[C{=}C]_{50\%}$-TAPH-COFs	460	23.1	29.0	14	
$[C{=}C]_{75\%}$-TAPH-COFs	390	19.2	28.7	16	
$[C{=}C]_{100\%}$-TAPH-COFs	310	19.2	28.5	22	
TH-COF-1	684	49.3	31	31	25
COF-LZU1	382	21.3	28	7	
COF_{DT}	2597	22.9	20.0	15.5	This work
COF_{DT}-Zn-NH_2	1180	32.5	25.1	153	

[a] The uptake was measured at 298 K and 1 bar.

[b] Determined by IAST theory for a 15/85 CO_2/N_2 mixture at 298 K and 1 bar.

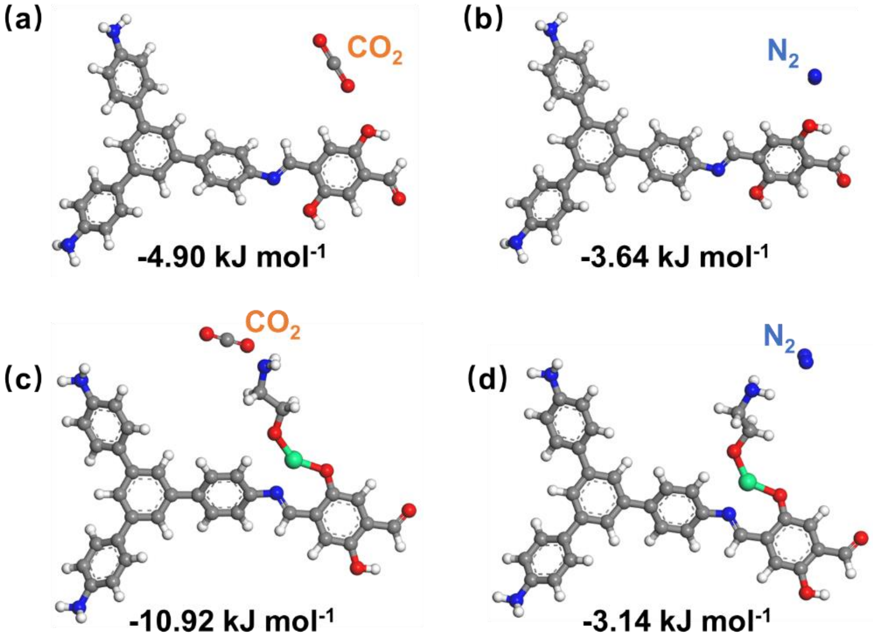

Figure S10. DFT-optimized stable adsorption configurations and binding energies of CO_2 and N_2 molecules on representative structural units of (a, b) COF_{DT} and (c, d) COF_{DT}-Zn-NH_2. (Gray, white, blue, red, and green atoms represent C, H, N, O, and Zn, respectively.)

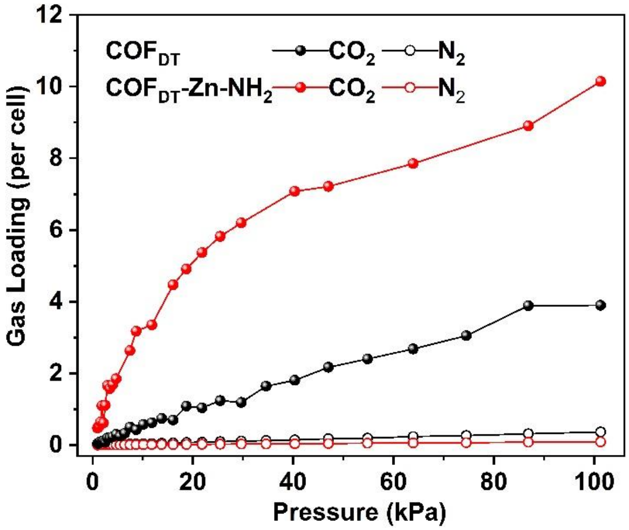

Figure S11. CO_2 and N_2 adsorption isotherms of COF_{DT} and COF_{DT}-Zn-NH_2 at 298 K obtained from GCMC simulations.

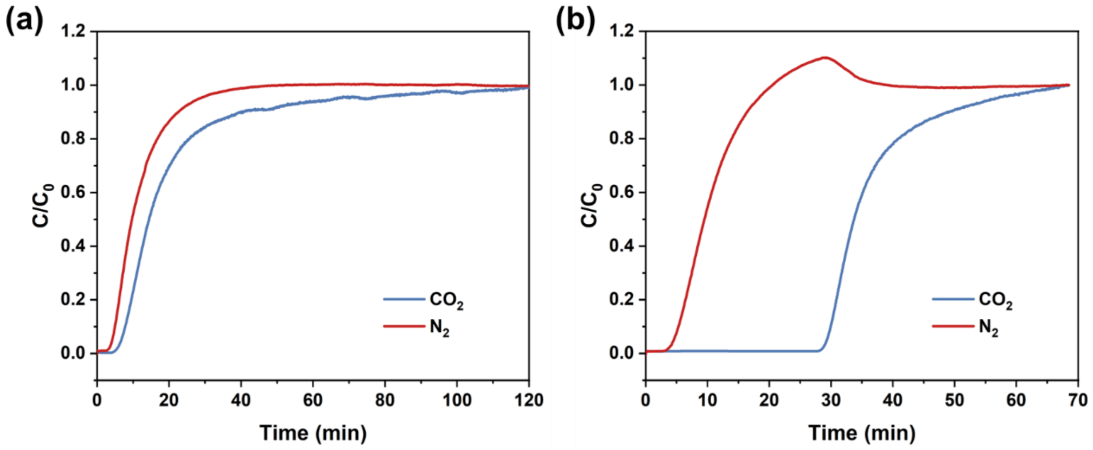

Figure S12. Breakthrough curves of the CO_2/N_2 (15:85, v/v) mixture at 298K for (a) COF_{DT} and (b) COF_{DT}-Zn-NH_2.

As nitrogen is a weakly adsorbed component, its quantitative analysis is subject to measurement errors. Nevertheless, compared with the pristine COF_{DT}, MSP modification substantially enhances the separation selectivity between CO_2 and N_2.

References

(1) Riazi, M. R.; Khan, A. R. A Thermodynamic Model for Gas Adsorption Isotherms. *J. Colloid Interface Sci.* **1999**, *210* (2), 309–319.

(2) Myers, A. L.; Prausnitz, J. M. Thermodynamics of Mixed-Gas Adsorption. *AIChE J.* **1965**, *11* (1), 121–127.

(3) Suo, X.; Cui, X.; Yang, L.; Xu, N.; Huang, Y.; He, Y.; Dai, S.; Xing, H. Synthesis of Ionic Ultramicroporous Polymers for Selective Separation of Acetylene from Ethylene. *Adv. Mater.* **2020**, *32* (29), 1907601.

(4) Gui, B.; Liu, X.; Cheng, Y.; Zhang, Y.; Chen, P.; He, M.; Sun, J.; Wang, C. Tailoring the Pore Surface of 3D Covalent Organic Frameworks via Post-synthetic Click Chemistry. *Angew. Chem. Int. Ed.* **2022**, *61* (2).

(5) Frisch, M. J.; Trucks, G. W.; Schlegel, H. B.; Scuseria, G. E.; Robb, M. A.; Cheeseman, J. R.; Scalmani, G.; Barone, V.; Petersson, G. A.; Nakatsuji, H.; Li, X.; Caricato, M.; Marenich, A. V.; Bloino, J.; Janesko, B. G.; Gomperts, R.; Mennucci, B.; Hratchian, H. P.; Ortiz, J. V.; Izmaylov, A. F.; Sonnenberg, J. L.; Williams-Young, D.; Ding, F.; Lipparini, F.; Egidi, F.; Goings, J.; Peng, B.; Petrone, A.; Henderson, T.; Ranasinghe, D.; Zakrzewski, V. G.; Gao, J.; Rega, N.; Zheng, G.; Liang, W.; Hada, M.; Ehara, M.; Toyota, K.; Fukuda, R.; Hasegawa, J.; Ishida, M.; Nakajima, T.; Honda, Y.; Kitao, O.; Nakai, H.; Vreven, T.; Throssell, K.; Montgomery, J. A., Jr.; Peralta, J. E.; Ogliaro, F.; Bearpark, M. J.; Heyd, J. J.; Brothers, E. N.; Kudin, K. N.; Staroverov, V. N.; Keith, T. A.; Kobayashi, R.; Normand, J.; Raghavachari, K.; Rendell, A. P.; Burant, J. C.; Iyengar, S. S.; Tomasi, J.; Cossi, M.; Millam, J. M.; Klene, M.; Adamo, C.; Cammi, R.; Ochterski, J. W.; Martin, R. L.; Morokuma, K.; Farkas, O.; Foresman, J. B.; Fox, D. J. Gaussian˜16 Revision C.01, 2016.

(6) Adamo, C.; Barone, V. Toward Reliable Density Functional Methods without Adjustable Parameters: The PBE0 Model. *J. Chem. Phys.* **1999**, *110* (13), 6158–6170.

(7) Burke, K.; Ernzerhof, M.; Perdew, J. P. The Adiabatic Connection Method: A Non-Empirical Hybrid. *Chem. Phys. Lett.* **1997**, *265* (1), 115–120.

(8) Grimme, S.; Ehrlich, S.; Goerigk, L. Effect of the Damping Function in Dispersion Corrected Density Functional Theory. *J. Comput. Chem.* **2011**, *32* (7), 1456–1465.

(9) Wadt, W. R.; Hay, P. J. Ab Initio Effective Core Potentials for Molecular Calculations. Potentials for Main Group Elements Na to Bi. *J. Chem. Phys.* **1985**, *82* (1), 284–298.

(10) Hay, P. J.; Wadt, W. R. Ab Initio Effective Core Potentials for Molecular Calculations. Potentials for K to Au Including the Outermost Core Orbitals. *J. Chem. Phys.* **1985**, *82* (1), 299–310.

(11) Hay, P. J.; Wadt, W. R. Ab Initio Effective Core Potentials for Molecular Calculations. Potentials for the Transition Metal Atoms Sc to Hg. *J. Chem. Phys.* **1985**, *82* (1), 270–

283.
(12) Grev, R. S.; Schaefer, H. F., III. 6-311G Is Not of Valence Triple-zeta Quality. *J. Chem. Phys.* **1989**, *91* (11), 7305–7306.
(13) Boys, S. F.; and Bernardi, F. The Calculation of Small Molecular Interactions by the Differences of Separate Total Energies. Some Procedures with Reduced Errors. *Mol. Phys.* **1970**, *19* (4), 553–566.
(14) Adams, D. j. Grand Canonical Ensemble Monte Carlo for a Lennard-Jones Fluid. *Mol. Phys.* **1975**, *29* (1), 307–311.
(15) Sun, H. COMPASS: An Ab Initio Force-Field Optimized for Condensed-Phase Applications-Overview with Details on Alkane and Benzene Compounds. *J. Phys. Chem. B* **1998**, *102* (38), 7338–7364.
(16) Ewald, P. P. Die Berechnung Optischer Und Elektrostatischer Gitterpotentiale. *Ann. Phys.* **1921**, *369* (3), 253–287.
(17) Darden, T.; York, D.; Pedersen, L. Particle Mesh Ewald: An N·log(N) Method for Ewald Sums in Large Systems. *J. Chem. Phys.* **1993**, *98* (12), 10089–10092.
(18) Rappe, A. K.; Goddard, W. A. I. Charge Equilibration for Molecular Dynamics Simulations. *J. Phys. Chem.* **1991**, *95* (8), 3358–3363.
(19) Wang, Y.; Kang, C.; Zhang, Z.; Usadi, A. K.; Calabro, D. C.; Baugh, L. S.; Yuan, Y. D.; Zhao, D. Evaluation of Schiff-Base Covalent Organic Frameworks for CO_2 Capture: Structure–Performance Relationships, Stability, and Performance under Wet Conditions. *ACS Sustain. Chem. Eng.* **2022**, *10* (1), 332–341.
(20) Stegbauer, L.; Hahn, M. W.; Jentys, A.; Savasci, G.; Ochsenfeld, C.; Lercher, J. A.; Lotsch, B. V. Tunable Water and CO_2 Sorption Properties in Isostructural Azine-Based Covalent Organic Frameworks through Polarity Engineering. *Chem. Mater.* **2015**, *27* (23), 7874–7881.
(21) Huang, N.; Krishna, R.; Jiang, D. Tailor-Made Pore Surface Engineering in Covalent Organic Frameworks: Systematic Functionalization for Performance Screening. *J. Am. Chem. Soc.* **2015**, *137* (22), 7079–7082.
(22) Huang, N.; Chen, X.; Krishna, R.; Jiang, D. Two-Dimensional Covalent Organic Frameworks for Carbon Dioxide Capture through Channel-Wall Functionalization. *Angew. Chem. Int. Ed.* **2015**, *54* (10), 2986–2990.
(23) Pyles, D. A.; Crowe, J. W.; Baldwin, L. A.; McGrier, P. L. Synthesis of Benzobisoxazole-Linked Two-Dimensional Covalent Organic Frameworks and Their Carbon Dioxide Capture Properties. *ACS Macro Lett.* **2016**, *5* (9), 1055–1058.
(24) Zhao, S.; Dong, B.; Ge, R.; Wang, C.; Song, X.; Ma, W.; Wang, Y.; Hao, C.; Guo, X.; Gao, Y. Channel-Wall Functionalization in Covalent Organic Frameworks for the Enhancement of CO_2 Uptake and CO_2/N_2 Selectivity. *RSC Adv.* **2016**, *6* (45), 38774–

38781.

(25) Wang, L.; Dong, B.; Ge, R.; Jiang, F.; Xiong, J.; Gao, Y.; Xu, J. A Thiadiazole-Functionalized Covalent Organic Framework for Efficient CO_2 Capture and Separation. *Microporous Mesoporous Mater.* **2016**, *224*, 95–99.
